# Fiber Bragg Grating Sensors for Underwater Vibration Measurement: Potential Hydropower Applications

**DOI:** 10.3390/s21134272

**Published:** 2021-06-22

**Authors:** Oscar de la Torre, Ignazio Floris, Salvador Sales, Xavier Escaler

**Affiliations:** 1 Barcelona Fluids & Energy Lab, Universitat Politécnica de Catalunya, 08028 Barcelona, Spain; xavier.escaler@upc.edu; 2 ITEAM, Universitat Politecnica de Valencia, 46022 Valencia, Spain; igflo@upv.es (I.F.); ssales@upv.es (S.S.)

**Keywords:** fluids, hydroelectric power generation, optical fiber sensors, vibration measurement

## Abstract

The present paper assesses the performance and characteristics of fiber Bragg grating sensors, with a special interest in their applications in hydraulic machinery and systems. The hydropower industry is turning to this technology with high expectations of obtaining high quality data to validate and calibrate numerical models that could be used as digital twins of key assets, further strengthening the sector’s relevant position within industry 4.0. Prior to any validation, fiber Bragg grating sensors’ ability to perform well underwater for long periods of time with minimal degradation, and their ease of scalability, drew the authors´ attention. A simplified modal analysis of a partially submerged beam is proposed here as a first step to validate the potential of this type of technology for hydropower applications. Fiber Bragg grating sensors are used to obtain the beam’s natural frequencies and to damp vibrations under different conditions. The results are compared with more established waterproof electric strain gauges and a laser vibrometer with good agreement. The presence of several sensors in a single fiber ensures high spatial resolution, fundamental to precisely determine vibration patterns, which is a main concern in this industry. In this work, the beam’s vibration patterns have been successfully captured under different excitations and conditions.

## 1. Introduction

The larger weight of intermittent and unpredictable renewable energy sources (solar, wind) in the current global energy mix, forces hydraulic turbines to operate discontinuously and frequently outside their best efficiency range to balance out. This is the fundamental reason behind the EU H2020 funded project called AFC4Hydro [1]. In this project, an active flow control system will be developed to guarantee that a turbine works safely at off-design conditions. This new system will be tested in a real industrial environment with a clear intention of reaching the market. To guarantee the safety of an operating hydraulic turbine, it is required to monitor a multitude of variables (structural, flow, electrical, etc.) and build predictive models for risk assessment. In AFC4Hydro, a structural health monitoring system will be implemented, combining experimental tests and numeric models in a sort of digital twin for different environments (laboratory and real power plant). Building digital twins of key engineering infrastructures is growing in popularity, both in research and in industrial environments for design and/or operation phases. Bridging the physical (sensors) and the numeric world (models) gives the users insights that neither of them separately can [2]. Monitoring a complex engineering infrastructure, such as a hydraulic turbine, implies using a variety of sensors, which will be working in harsh conditions (water, humidity, rotating components, electromagnetic interference, etc.). At the same time, it is essential to obtain as much experimental data as possible to build accurate numeric models. Ideally, this data should be well spatially and time-distributed, i.e., should cover different locations within the turbine and monitor long periods of time. Fiber-optic sensors (FOSs) are extremely suitable for such purposes. This innovative technology, extremely sensitive to temperature and strain variations [3], has been developed rapidly in recent decades [4] thanks to a multitude of cutting-edge advantages [5]. In particular, fiber Bragg gratings (FBGs) are by far the most widely used FOS [6] by virtue of their cost-effectiveness [7]. In this context, FBG sensors seemed a perfect candidate [8,9] to monitor a hydraulic turbine due to their:

- resistance to humidity (can be used underwater during long periods of time);
- corrosion resistance;
- compactness, small size, and light weight;
- immunity to electromagnetic interference (EMI);
- embedding capability;
- intrinsic safety (no electricity required for the sensor operation);
- bespoke spatial resolution;
- experimental setup simplification.

It is well-known that the dynamic characteristics of a structure submerged in water is greatly modified due to the added mass of the surrounding fluid [10,11]. In particular, natural frequencies, damping, and modes of vibration may change due to this phenomenon. However, added mass is a complex phenomenon which depends on many parameters, and sometimes only experimental data can be reliably used to calculate it. Experimentalists have been using special waterproof instrumentation, which is significantly more expensive than its non-sealed counterpart and still requires the same setup as in dry conditions. FBG sensors present several advantages over this type of instrumentation but, in particular, the authors are especially interested in their performance underwater and the spatial resolution. Before using FBG sensors in industrial turbines, the authors designed a simplified but representative laboratory-scale experiment to test the performance of this technology. Impact tests impose several challenges for a sensing technology that a static test does not. First, it should be able to read very different amplitude levels (typical of a sudden hit) and second, it should be capable of acquiring at a rate high enough to capture the desired frequencies. However, this test has not been chosen because it is a tool to validate a technology, it has been selected because this type of test is very common in the hydropower field where dynamic testing is required to study most fluid structure interaction phenomena.

This paper aims to validate the use of FBG sensors for potential hydropower applications. Pros and cons of using FBG sensors in this industry are summarized.

## 2. Methodology

### 2.1. Test Introduction

Structural dynamics testing (SDT) provides an effective way to characterize the dynamic behaviors of structures or more complex systems. SDT comprises a wide range of tests and amongst them modal testing (MT) is probably the most popular. MT refers to a test where a structure is vibrated with a known excitation under controlled conditions [12]. The main objective of this test is to obtain a mathematical description of the dynamic behavior of the structure under study. The complexity of this “description” varies greatly from test to test and is mainly determined by the subsequent requirements of the experimentalist. Some may require a complete model within a frequency range, some others may only need an estimate of a specific natural frequency. Regardless of the type of outcome required, MT is nowadays fully integrated within the design phase of virtually any engineering infrastructure ([13,14,15,16]).

Two of the most popular ways to vibrate the structure are by means of an impact hammer and a shaker [17]. An impact hammer is a hammer with a dynamic force sensor on its tip that allows for precise monitoring of the input force. The force is input as a sudden hit, which in the frequency domain becomes a constant force within a relatively wide frequency range. A shaker is an electro-mechanical actuator that is in contact with the structure under study and that can be fed with different excitation signals (fixed frequency sinusoidal, chirps, white noise). The structure’s response is then recorded by sensors such as accelerometers, displacement sensors, vibrometers, which produce an electric signal proportional to a certain physical magnitude.

### 2.2. Fiber Bragg Grating Sensors

**Figure 1:**
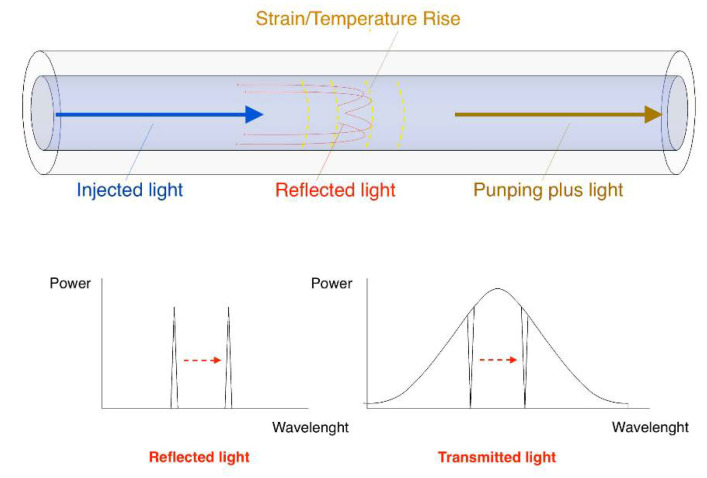
Sensing principle of fiber Bragg grating.

FBG sensors are Bragg reflectors, fabricated by laterally exposing the core of an optical fiber to an intense laser light with a periodic pattern in order to permanently increase its refractive index [6]. Such fixed index modulation is a grating and has a period that depends on the exposure pattern and on the temperature and longitudinal strain. Since an FBG transmits some wavelengths and reflects others, corresponding to its wavelength’s peak (see Figure 1), by tracking it, it is possible to sense the temperature and strain variations which the grating is subject to. The mathematical relationship between the shifts of the Bragg wavelength peak and the temperature or strain changes can be written as [18]:

(1) $$\frac{\Delta \lambda }{\lambda_{0}}=\left(1-p_{eff}\right)\Delta \varepsilon +\left(\alpha +\xi \right)\Delta T$$

where Δ*λ*, *λ_0_*, Δ*ε*, and Δ*T* are the wavelength variation, the wavelength value and the variation of strain and temperature, respectively. While *p_eff_*, *α* and *ξ* are, the photo-elastic coefficient, the thermal expansion coefficient, and the thermal-optic coefficient, respectively.

Fiber Bragg gratings are the most widely used FOSs, well-recognized as extremely sensitive strain and temperature single-point sensors, and employed in a multitude of engineering applications [19,20,21,22,23]. The principal reasons behind this extensive use are:

- the large sensing length (~km);
- the low cost;
- the high strain sensing accuracy (~1 με);
- the ability to perform dynamic sensing with high frequency sampling rates (~kHz).

### 2.3. Experimental Setup

**Figure 2:**
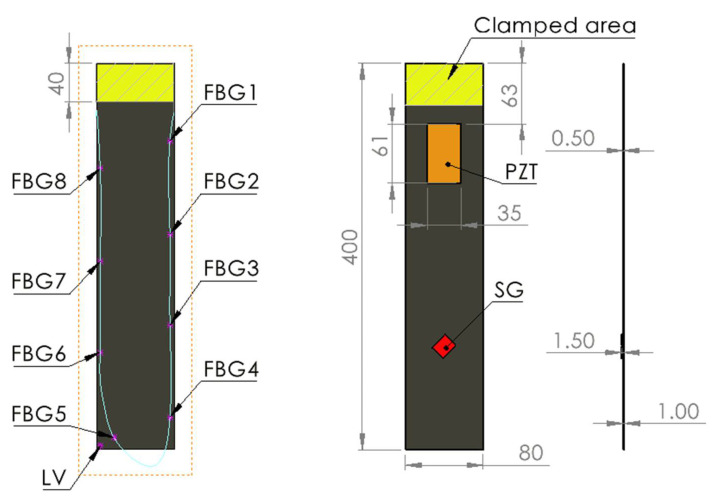
Front, back, and lateral view of the plate with the different sensing technologies used and the main dimensions in mm (FBG = fiber Bragg grating sensor, SG = strain gauge, PZT = piezoelectric patch, LV = laser vibrometer).

In order to assess FBG sensors’ ability to work underwater and compare it against other established electrical sensing (waterproof) technologies, several of these technologies were tested. All of them on the same test sample: an 80 mm wide, 400 mm long, and 1 mm thick stainless-steel plate. An array of eight FBG sensors (manufactured internally at ITEAM facilities, Valencia, Spain) was installed on one of the faces of the plate, approximately following its perimeter (Figure 2). The array consisted of a single cable line with the eight sensors embedded in it evenly spaced. All sensors except FBG 5 were installed aligned with the beam’s longitudinal axis to measure the bending-induced strain. FBG 5, which was located in the arc section of the array ended up oriented at approximately 45° because the minimal bending radius optical fibers can withstand is relatively large. The line diameter was 250 micrometers, which made its installation challenging. Only the sensors’ locations were glued to the plate using a two-phase glue. Between two glued points, the cable had some slack to allow for the plate deformation during the test. An extra FBG sensor was used to monitor the temperature differences during the test to compensate for temperature-induced strain if necessary. However, this phenomenon seemed negligible, given the dynamic and rapid nature of the tests described in this paper. Therefore, Equation (1) has been simplified to:

(2) $$\frac{\Delta \lambda }{\lambda_{0}}=\left(1-p_{eff}\right)\Delta \varepsilon$$

A rosette electrical strain gauge (SG) was also installed on the opposite side of the plate in its neutral axis using a typical cyanoacrylate-based glue. A three-wire rosette SG was used to increase its sensitivity and compensate for leadwire temperature changes. The three SGs stacked in a rosette configuration were oriented at 0°, 45°, and 90°, where 0° was aligned with the plate’s longitudinal axis. The SGs were coated with an epoxy resin to make them waterproof. Additionally, a single point laser doppler vibrometer (LV) was also used to monitor the plate’s response. This contactless technology is especially useful for applications of difficult access and for vibration tests due to its high sampling rate. Figure 2 schematically shows the different views of the plate with the different technologies installed on it.

**Figure 3:**
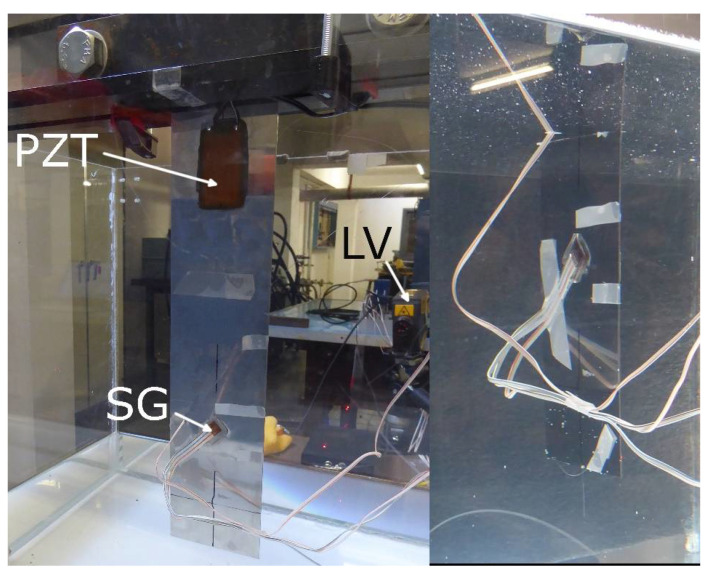
Cantilever beam above an empty (left) and a partially filled (right) plexiglass tank.

A very simple test procedure was designed to perform the comparison. The plate was set in a cantilever configuration using clamping fixtures along 40 mm of length above a cubic plexiglass tank (approximately 500 mm × 500 mm × 500 mm), vertically oriented and with its free end on the bottom (Figure 3). Several impacts were performed in this configuration to extract the plate’s natural frequencies. Then, the plate was excited at fixed frequencies around some of these natural frequencies. A piezoelectric (PZT) patch (PI Ceramic GmbH, Lederhose, Germany) was used to excite the structure using ±100 V sinusoidal signals of different frequencies obtained from an amplified signal generator. The PZT patch was glued on the plate’s surface using a two-phase epoxy glue. Then, the plexiglass tank was partially filled with water up to a fixed level where 173 mm of the beam was underwater. The impacts and the fixed sine frequency excitations were repeated under these new conditions.

FBG data was acquired at a sampling rate of 1 kHz using a Dynamic Optical Sensing Interrogator sm130, manufactured by Micron Optics. The accuracy of the dynamic data decreases at high-frequency acquisition; however, even in these conditions, FBG sensors are still capable of achieving a strain resolution of few microstrains. SGs and LV signals were acquired simultaneously at 2.5 kHz using a compatible acquisition system. A 30 s time signal was acquired with both systems. Four impacts were recorded for both an empty and a partially filled tank cases. For the fixed frequency sinusoidal excitation cases, also a 30 s signal was acquired. The excitation frequencies were determined based on the results of the previous impact test.

### 2.4. Theoretical Model

The main advantages of using a cantilever plate as a test sample are: (i) the simple geometry and configuration and (ii) the existence of analytical formulas to obtain its natural frequencies. If we focus on bending modes, the natural frequencies follow [24]:

(3) $$f_{i}=\frac{\lambda_{i}}{2\pi L^{2}}\sqrt{\frac{EI}{\rho A}}$$

where *E* is the material Young’s modulus, ρ its density, L is the effective length of the beam, A is the cross section of the beam, and I is its area moment of inertia about the width axis, respectively. The figure $\lambda_{i}$ is a nondimensional parameter that identifies the mode of vibration. Table 1 shows its values for the three first modes for a cantilever configuration:

## 3. Results

### 3.1. Impact Tests

#### 3.1.1. Dry Conditions

**Figure 4:**
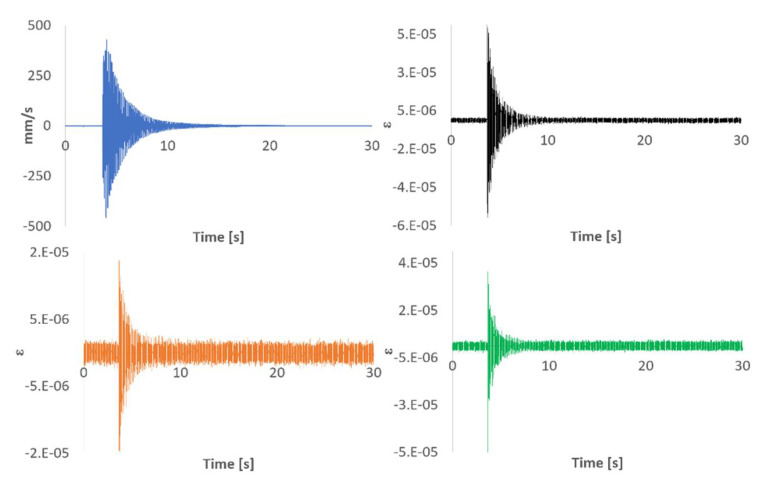
Impact time signal responses measured with LV (top left), SG 0º (top right), SG 90º (bottom left), and SG 45º (bottom right).

**Figure 5:**
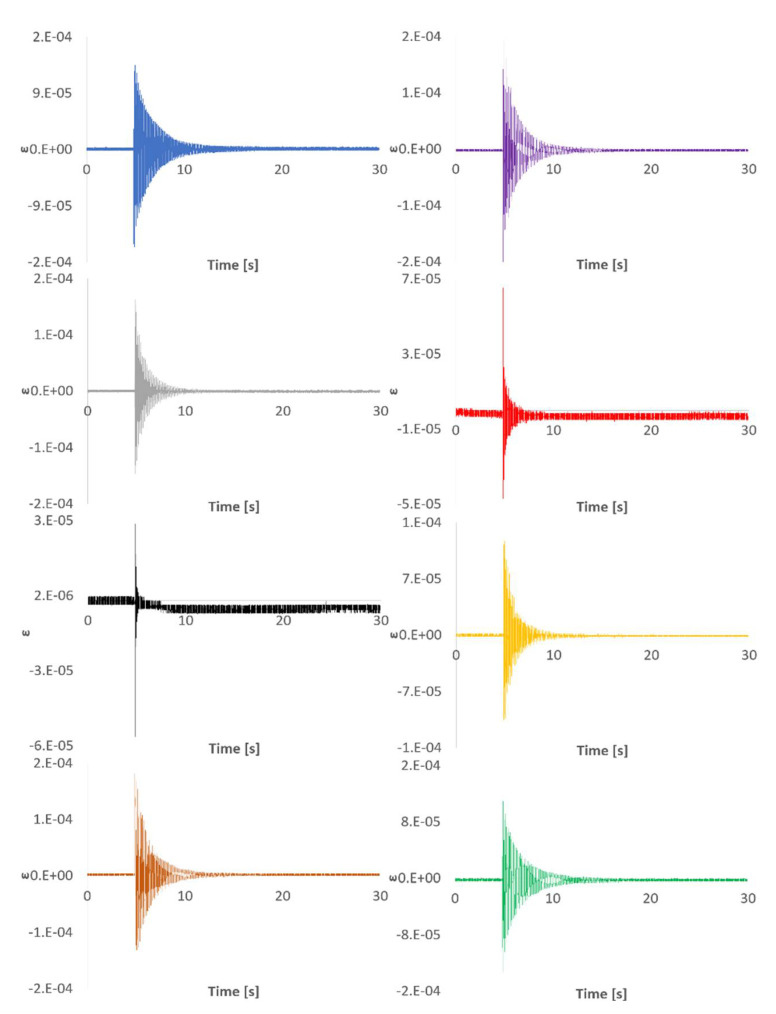
Impact time signal responses measured with FBG sensors: FBG 1 (blue), FBG 2 (purple), FBG 3 (grey), FBG 4 (red), FBG 5 (black), FBG 6 (yellow), FBG 7 (brown), and FBG 8 (green).

As commented above, the beam was hit and the responses recorded using both the triaxial SG, the LV, and the FBG sensors. Figure 4 shows an example of the 30 s time signals recorded with the SG and the LV. Capturing long enough time signals increases the resolution when working in the frequency domain. An analogous graph plotting the beam´s response measured with the FBG sensors is shown in Figure 5. For the sake of space, the different signals have been overlaid in the rest of the figures of this document.

**Figure 6:**
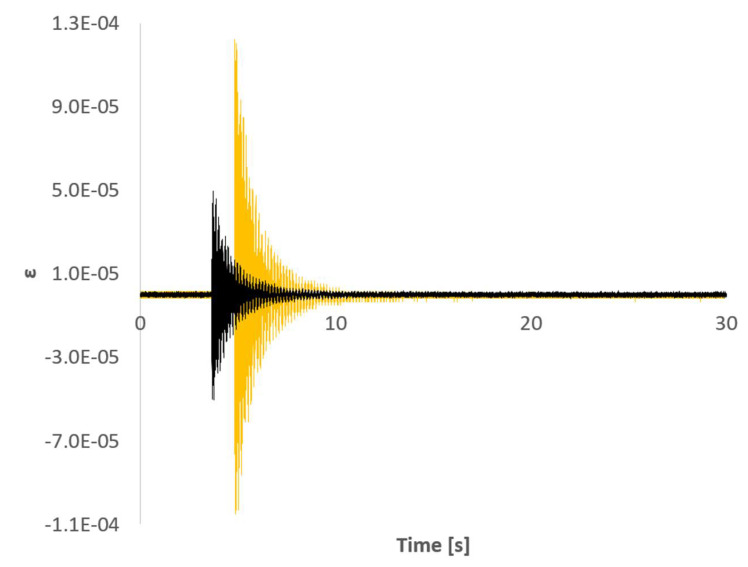
Time signal responses of SG 0º (black) and FBG6 (yellow).

Due to the distribution of the FBG sensors on the beam´s surface and the location of the impact point (not in the neutral axis of the beam) those sensors between the clamped area and the SG location read higher strain values than any of the directions of the SG. The quality of the impact read by FBG 5 was significantly lower than that of the rest of the sensors due to its location and different orientation. Figure 4 and Figure 5 show that both sampling rates (2.5 kHz and 1 kHz) are capable of capturing the typical logarithmic decrement shape of the response. The distance from the clamped region to FBG6 and to SG is very similar (see Figure 2). Moreover, FBG6 and SG 0º are both parallel to the plate’s longitudinal axis. Therefore, strain amplitudes captured by both sensors should be similar. However, as shown in Figure 6, FBG6 strain amplitudes are higher than SG 0º. This is probably due to a better longitudinal alignment of the FBG array compared to the rosette SG. The fact that FBG sensors come embedded in a line makes it easier to accurately orientate them in specific directions.

**Figure 7:**
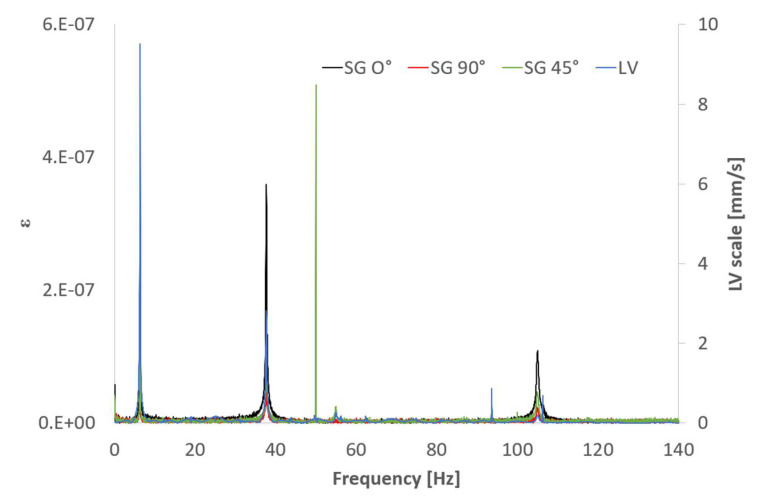
FFT of the SG and LV signals.

The use of the Fast Fourier Transform (FFT) converts the time domain to the frequency domain. The FFT is defined by Equation (4):

(4) $$X\left(f\right)=\int_{-\infty }^{\infty }x\left(t\right)e^{-j2\pi f}dt$$

where x(t) is the signal in the time domain and *f* is the frequency. Figure 7 shows the FFT of the impact responses acquired with the LV and the SGs. The frequency range has been selected to visualize the first three bending frequencies. Together with the three desired frequencies, a sharp peak appears at 50 Hz in the SG signals, which is characteristic of electrical noise. Note that due to the impact location (close to a nodal line), the third bending peak is significantly lower than the previous two.

**Figure 8:**
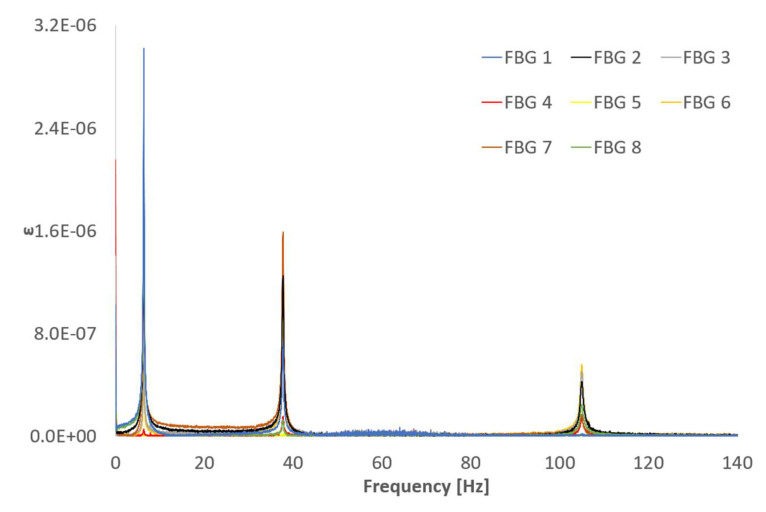
FFT of the FBG sensors signals.

Similarly, Figure 8 shows the frequency content of the FBG’s responses. It is evident that FBG’s signals are significantly cleaner than the SG’s. This is one of the main advantages of fiber optics—their insensibility to noise sources, such as electromagnetic interferences, which would be extremely useful in industrial environments such as hydropower stations. It is important to remember here that the SGs and LV (Figure 7) have been sampled at a 2.5 times higher sampling rate than the FBGs (Figure 8). Therefore, any visual comparison of noise floor level could be misleading. Theoretically, the noise would be distributed along the frequency range determined by the Nyquist frequency; hence, a higher sample rate would apparently produce a lower noise floor level because the same amount of noise would be distributed along a wider frequency range.

**Figure 9:**
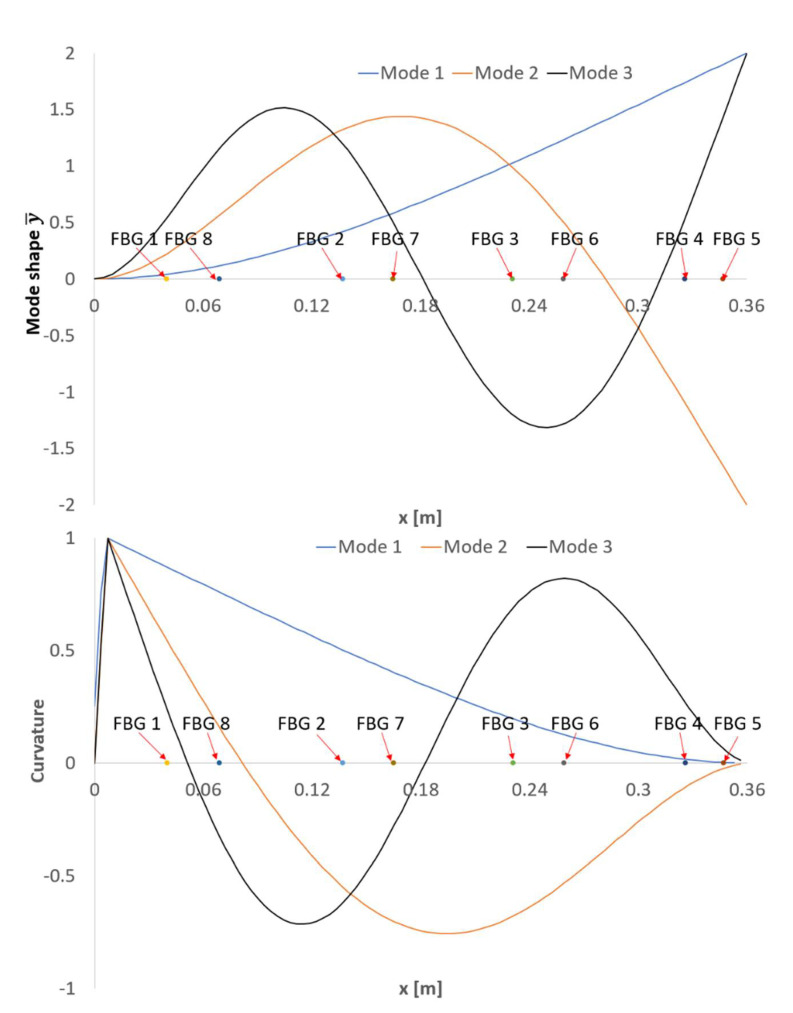
The beam´s first three bending modes with the location of the FBG sensors: displacement field (above), strain field (below).

The natural frequencies obtained for all the sensors are consistent and similar to the ones obtained using the theoretical approximation (3) shown in Table 2. The corresponding errors relative to the theoretical values are summarized in Table 3. The different alignment of FBG 5 makes it unsuitable to detect any bending mode. The bending strain field is proportional to the deflection curve and, hence, to the second derivative of the displacement. This is very important to consider when performing MT with strain sensors (like FBG and SG) to locate the sensors accordingly to maximize their response. For instance, FBG 1 is located in an area of low curvature for the third bending mode and this is why the frequency is not captured. Figure 9 shows the first three bending mode shapes and their second derivatives.

An unscaled damping factor calculation for each of the three bending modes can be used to compare the shape of the frequency peaks among the different sensors and hence the similarities of the signals. The damping factors of the system, $\zeta_{i}$, can be calculated using the half-power method for each of the peaks under study. This is a very simple method, which assumes well-separated mode shapes and it is extremely sensitive to small errors in the estimation of the frequencies. This sensitivity to the frequency peak shape is precisely the reason why this method has been selected, as it can be related to the level of similarity of the frequency signal around the natural frequencies. The results shown in Table 4 are very consistent through the different technologies for the modes under study.

#### 3.1.2. Partially Submerged

**Figure 10:**
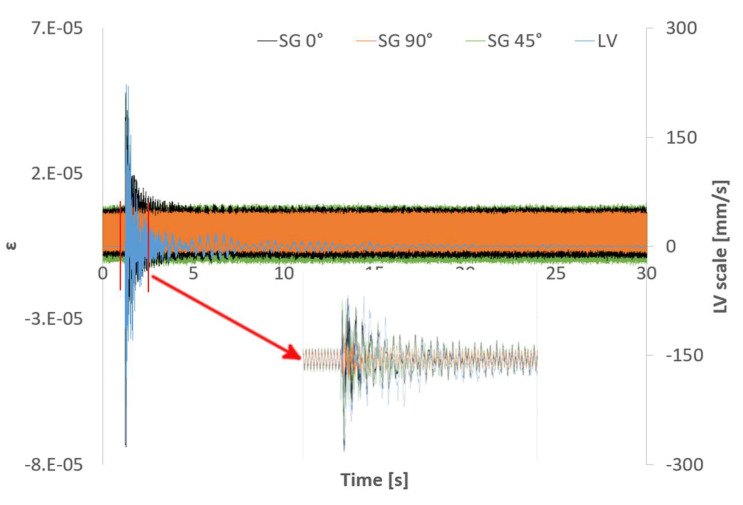
Time signals for an impact response for the SGs and the LV in partial submergence conditions. A zoomed view of the impact is also shown.

**Figure 11:**
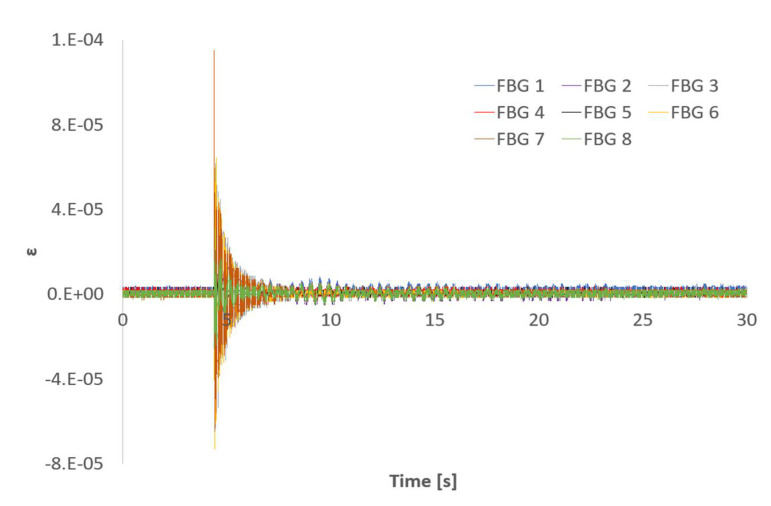
Time signals for an impact response for FBG sensors in partial submergence conditions.

The dynamics of submerged (or partially submerged) structures is typically more complex than its dry counterpart because the structure and the fluid combine into a coupled system resulting in a fluid structure interaction (FSI). The fluid adds inertia and viscous damping to the structure and modifies its modes of vibration. Moreover, the fluid adds noise to the sensors signals and their analysis becomes more challenging. Figure 10 and Figure 11 shows the SGs and LV responses and the FBG sensors responses to an impact in partially submerged conditions, respectively. Comparing the time signals to those shown in Figure 4 and Figure 5, the increase on the noise floor for the SGs signals and the viscous damping added by the fluid in the LV and the FBG signals are evident. The significant increase in the noise floor level for the SG signals is due to electrical noise, probably due to a leakage to ground problem in the instrumentation setup. Fortunately, FBG sensors are immune to this type of noise. However, the higher noise floor compared to the dry case in Figure 11 is due to the presence of the surrounding fluid. When submerged, the movement of the plate entrains the surrounding fluid. Since the fluid is contained in a limited tank, the timescale to damp out the fluid movement is significantly longer than the one shown by the plate, which adds noise to the signals.

Similarly, we can find the natural frequencies of the beam in these new conditions. Due to the inertia added by the fluid, the new natural frequencies will be lower and the frequency shift will be mode-dependent [25]. A coarse approximation could be extracted from the results in [26] where the added mass of totally submerged thin rectangular plates were calculated using potential flow theory. We could consider the contribution of the submerged part of the beam and add that fluid mass to Equation (3). This is of course a great simplification because the added mass effect is not linearly proportional to the submergence depth, it is in fact mode-shape-dependent because the areas of the beam with higher displacements contribute more to this phenomenon—unlike nodal lines, which do not contribute at all. Moreover, in partial submergence conditions, there exists a free surface effect, which has not been considered here. Table 5 shows the expected frequency values using this approximation.

The experimental values obtained for the different sensors in partial submergence conditions are summarized in Table 6. The results are very consistent for all sensor types and the natural frequencies are lower than those obtained in dry conditions, as expected. As observed, the approximation (Table 5) underpredicts the added mass effect especially for *f*_1w_ and overpredicts the effect for *f*_3w_. Similarly to the dry case, FBG 5 is unable to capture the different natural frequencies due to its different orientation. FBG 1 and FBG 8, which were not adequately located to capture the third bending mode, as seen before, do not show a frequency peak for *f_3w_.* Particularly under partial submergence, the peaks are highly damped due to the fluid.

**Figure 12:**
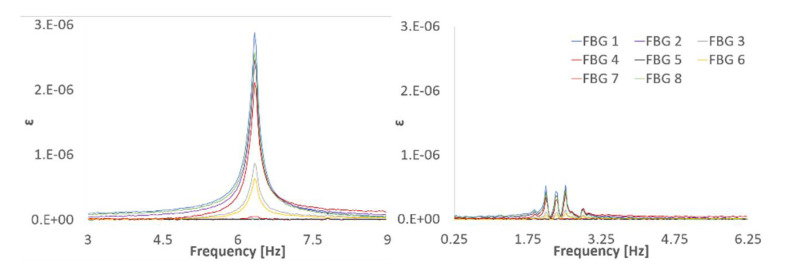
First bending peak comparison for: dry conditions (left), partially submerged (right).

Damping ratios are summarized in Table 7. As expected, the damping ratios are higher than those found in dry conditions. The variability of the results for all sensor types, particularly for $\zeta_{1}$, is evident. For modes with relatively large displacements, the movement of the fluid with respect to the structure induces submergence variations that results in a narrow frequency band excited instead of a clear peak (Figure 12). The half-power method to obtain the damping is very sensitive to any variation of the frequency and the shape of the peak; hence, the authors have highlighted in red the best-located sensors in Table 7 for each mode, which should produce the most accurate results. As expected, those sensors best located to capture a particular natural frequency are also the most reliable to obtain the damping ratio. In general, the results of FBG sensors are similar to those obtained with SGs or LV.

### 3.2. Fixed Frequency Excitation

One of the main advantages of FBG compared to other types of sensors is its scalability. For FBG manufacturers it is relatively simple to fabricate many sensors in a given fiber length; and for the test operators installing a single fiber, which can contain many sensors, this simplifies the experimental setup significantly (wiring, number of available channels in the acquisition system, etc.) and increases the spatial resolution. This is especially interesting when analyzing systems like hydraulic turbines, where many subcomponents coexist and can show very complex modes of vibration. Moreover, increasing the number of sensors reduces the (relative) importance of badly located sensors (as seen for FBG 1 for mode 3).

In our setup, there were 8 FBG sensors distributed around the perimeter of the beam. Considering the relatively simple shape of bending modes and assuming lock in conditions, that is, for excitation frequencies close to a beam´s natural frequency, the contribution to the beam vibration pattern of other modes different than the one associated to that specific natural frequency is negligible and the vibration is locked in to that single mode, we can visualize the three bending modes using the results obtained by the FBG sensors to highlight the benefits of increasing the spatial resolution.

A PZT patch (Figure 2) was used to excite the beam at different fixed frequencies. Using an FFT, we can obtain the magnitude of strain at each location and frequency. FFT is a complex number, and the imaginary component gives us information about the relative direction of the magnitude for a given reference.

**Figure 13:**
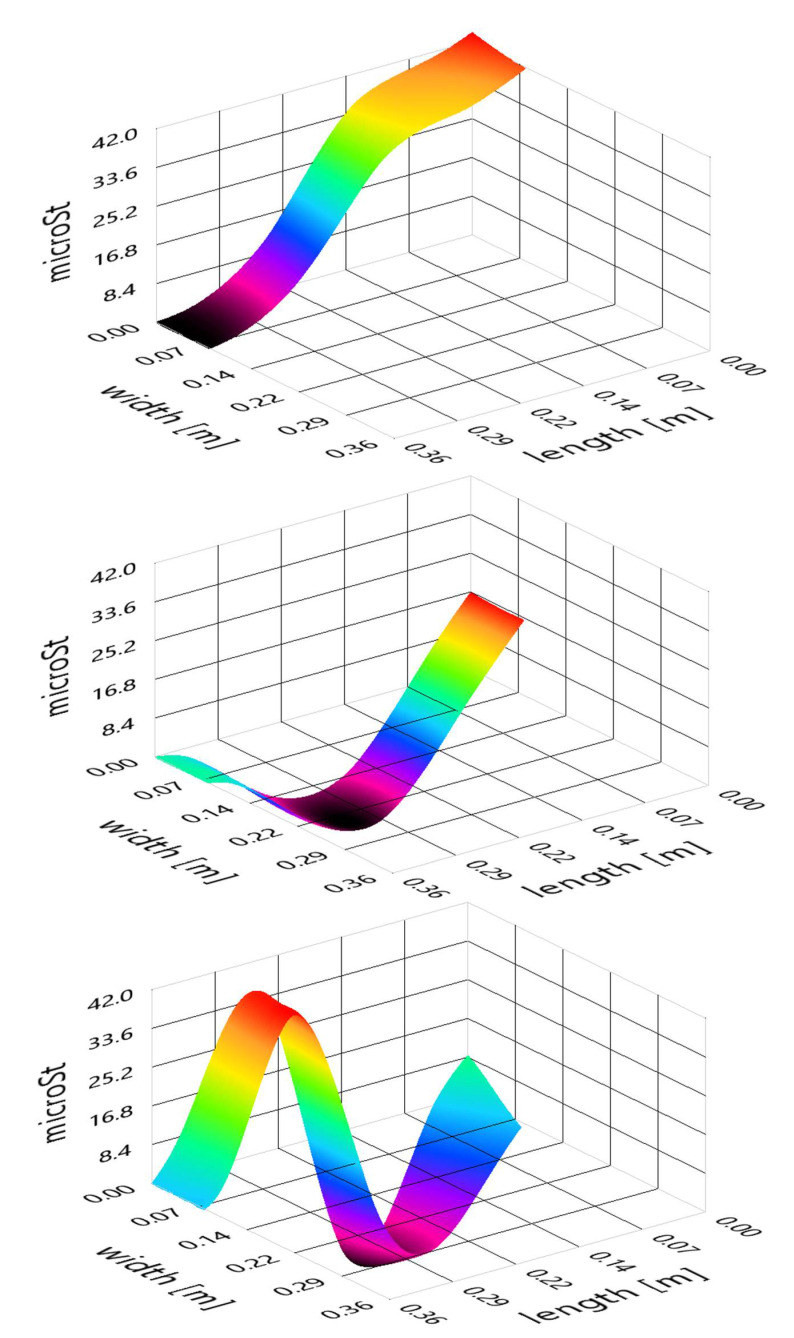
Beam´s experimental first three bending modes in air: first (top), second (middle), and third (bottom).

The beam has been discretized into a rectangular mesh with a 1 mm by 1 mm element size. Considering each FBG sensor location on the beam and the strain magnitude for the selected frequency, a two-dimensional interpolation over the whole mesh has been carried out. The three first bending modes of the beam in dry conditions have been plotted in Figure 13. As observed, the modes are very close to their theoretical counterpart shown in Figure 9 (bottom).

**Figure 14:**
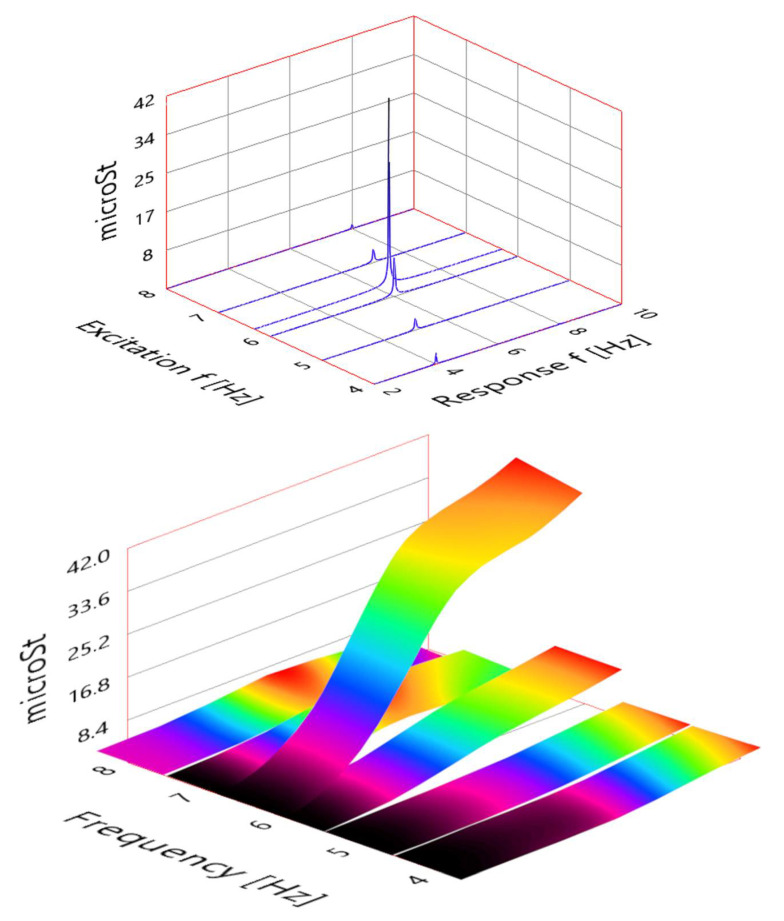
FBG 1 signal response (top) and beam mode shapes (bottom) for different frequency excitations.

This high spatial resolution offers several benefits, such as the possibility of verifying a crucial assumption. The lock-in condition can be checked by exciting the beam at fixed frequencies around a natural frequency and observing the vibration pattern. In this case, Figure 14 (bottom) shows the vibration pattern of the beam when excited at 4, 5, 6, 6.3, 7, and 8 Hz. The resonance is clearly visible at 6.3 Hz. It is interesting to observe how already at 7 and 8 Hz the shape of the vibration has completely changed and resembles more a second bending mode. A resonance phenomenon could be catastrophic for a hydraulic turbine, hence this could be very useful to determine resonance-free frequency regions to operate. Figure 14 (top) shows the variation of FBG 1 signal within the same frequency range.

**Figure 15:**
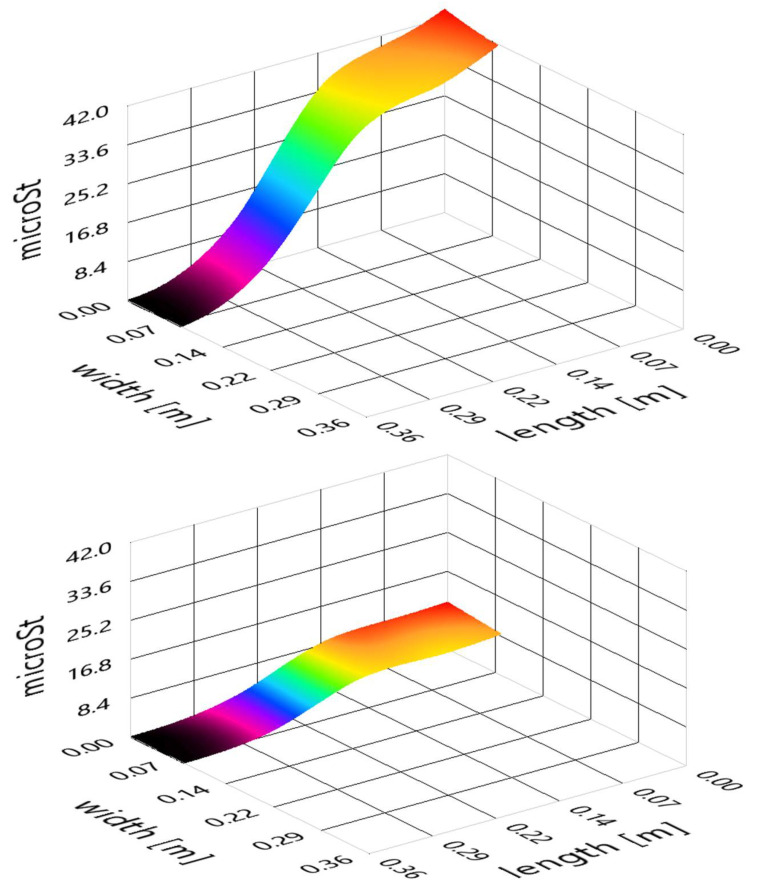
Beam first bending mode in air (top) and in water (bottom).

It has also been possible to study the mode variations due to the added mass of the fluid. Figure 15 shows the differences between the first bending mode in air and partially submerged in water. In hydropower applications it is usual to assume the modes are unaffected by the added mass for simplification, but in turbines with extremely small gap tolerances, even small changes in shape can have great importance. FBG technology shows itself capable of capturing those differences, performing well both in air and underwater.

## 4. Conclusions

In this document, the performance of FBG sensors in a modal analysis of a beam vibrating both in air and partially submerged in water has been assessed. This test was designed as a simplified first step to validate this technology for hydropower applications. FBG sensors have been capable to efficiently capture the first three bending natural frequencies of the beam in both conditions. The results were in good agreement with those from an electric (waterproof) SG and a LV. FBG signals also showed less electrical noise than those of SGs. The presence of several sensors in a single fiber proved very valuable to clearly plot the beam´s modes of vibration under different conditions without compromising the experimental setup. This is indeed a major benefit compared to other technologies in the field.

Based on the authors’ observations during the present work, the main advantages of this technology to penetrate in the hydropower industry would be:

- good performance underwater;
- ease of scalability to increase spatial resolution;
- high strain resolution;
- immunity to EMI.

However, limitations worth considering would be the fragility of the sensors of extremely small diameters and the fact that its installation requires trained staff.

## Figures and Tables

**Table 1:** $\lambda_{i}\;$ value for the first three bending modes.

Nondimensional Modal Parameter	Value
**$\lambda_{1}$**	1.875104
**$\lambda_{2}$**	4.694091
**$\lambda_{3}$**	7.854757

**Table 2:** Theoretical approximation for the three first bending frequencies.

	f_1_ (Hz)	f_2_ (Hz)	f_3_ (Hz)
Theory	6.23	39.06	109.36

**Table 3:** Relative error of the experimental values with respect to the theoretical approximation.

*Sensor*	Error_1_ (%)	Error_2_ (%)	Error_3_ (%)
*LV*	1.83	3.44	4.00
*SG 0°*	1.97	3.37	4.00
*SG 90°*	1.83	3.46	4.09
*SG 45°*	1.83	3.47	4.04
*FBG 1*	1.72	3.55	---
*FBG 2*	1.72	3.51	4.02
*FBG 3*	1.72	3.51	4.03
*FBG 4*	1.85	3.51	3.99
*FBG 5*	---	---	---
*FBG 6*	1.72	3.55	4.02
*FBG 7*	1.65	3.53	4.08
*FBG 8*	1.72	3.62	3.99

**Table 4:** Averaged damping factors and standard deviation for all sensors.

*Sensor*	$\zeta_{1}\;\left(\%\right)$	$\zeta_{2}\;\left(\%\right)$	$\zeta_{3}\;\left(\%\right)$
*LV*	1.15 ± 0.09	0.39 ± 0.02	0.28 ± 0.01
*SG 0°*	1.03 ± 0.07	0.38 ± 0.02	0.27 ± 0.01
*SG 90°*	0.99 ± 0.08	0.37 ± 0.02	0.28 ± 0.01
*SG 45°*	1.02 ± 0.04	0.38 ± 0.02	0.27 ± 0.02
*FBG 1*	1.03 ± 0.07	0.38 ± 0.01	---
*FBG 2*	1.02 ± 0.06	0.38 ± 0.02	0.27 ± 0.01
*FBG 3*	0.99 ± 0.08	0.38 ± 0.02	0.27 ± 0.01
*FBG 4*	1.23 ± 0.09	0.37 ± 0.01	0.28 ± 0.01
*FBG 5*	---	---	---
*FBG 6*	1.00 ± 0.06	0.38 ± 0.02	0.27 ± 0.01
*FBG 7*	0.95 ± 0.04	0.40 ± 0.00	0.27 ± 0.00
*FBG 8*	1.03 ± 0.07	0.36 ± 0.03	0.27 ± 0.01

**Table 5:** Theoretical approximation for the first three bending frequencies, partially considering added mass effect.

	f_1w_ (Hz)	f_2w_ (Hz)	f_3w_ (Hz)
Theory	3.63	22.78	63.78

**Table 6:** Experimental values for the first three bending frequencies in partial submergence conditions.

*Sensor*	f_1w_ (Hz)	f_2w_ (Hz)	f_3w_ (Hz)
*LV*	2.51	21.14	73.88
*SG 0°*	2.51	21.14	73.89
*SG 90°*	2.51	21.12	---
*SG 45°*	2.51	21.13	73.83
*FBG 1*	2.50	21.13	---
*FBG 2*	2.50	21.11	73.93
*FBG 3*	2.50	21.14	73.89
*FBG 4*	2.50	21.12	73.87
*FBG 5*	---	---	---
*FBG 6*	2.50	21.14	73.88
*FBG 7*	2.50	21.11	73.81
*FBG 8*	2.50	21.11	---

**Table 7:** Average damping rations and standard deviation for all sensors.

*Sensor*	$\zeta_{1}\;\left(\%\right)$	$\zeta_{2}\;\left(\%\right)$	$\zeta_{3}\;\left(\%\right)$
*LV*	4.59 ± 1.36	0.77 ± 0.05	0.76 ± 0.14
*SG 0°*	4.32 ± 1.54	0.73 ± 0.03	0.64 ± 0.06
*SG 90°*	6.62 ± 3.01	0.73 ± 0.02	---
*SG 45°*	3.66 ± 3.21	0.74 ± 0.05	0.63 ± 0.32
*FBG 1*	*2.60 ± 0.14*	0.72 ± 0.04	---
*FBG 2*	3.27 ± 0.62	0.75 ± 0.05	0.45 ± 0.19
*FBG 3*	3.82 ± 0.25	0.73 ± 0.03	0.55 ± 0.14
*FBG 4*	---	0.73 ± 0.03	0.68
*FBG 5*	---	---	---
*FBG 6*	5.09 ± 3.46	0.69 ± 0.02	*0.52 ± 0.11*
*FBG 7*	4.17	*0.70*	0.57
*FBG 8*	2.65 ± 0.17	0.72 ± 0.06	---

## Data Availability

Not applicable.
